# Use new PLGL-RGD-NGF nerve conduits for promoting peripheral nerve regeneration

**DOI:** 10.1186/1475-925X-11-36

**Published:** 2012-07-09

**Authors:** Qiongjiao Yan, Yixia Yin, Binbin Li

**Affiliations:** 1 Biomedical Materials and Engineering Center, Wuhan University of Technology, Wuhan, Peoples Republic of China

**Keywords:** RGD peptide, Nerve growth factor, Peripheral nerve, Nerve conduits, Nerve regeneration

## Abstract

**Background:**

Nerve conduits provide a promising strategy for peripheral nerve injury repair. However, the efficiency of nerve conduits to enhance nerve regeneration and functional recovery is often inferior to that of autografts. Nerve conduits require additional factors such as cell adhesion molecules and neurotrophic factors to provide a more conducive microenvironment for nerve regeneration.

**Methods:**

In the present study, poly{(lactic acid)-*co*-[(glycolic acid)-*alt*-(L-lysine)]} (PLGL) was modified by grafting Gly-Arg-Gly-Asp-Gly (RGD peptide) and nerve growth factor (NGF) for fabricating new PLGL-RGD-NGF nerve conduits to promote nerve regeneration and functional recovery. PLGL-RGD-NGF nerve conduits were tested in the rat sciatic nerve transection model. Rat sciatic nerves were cut off to form a 10 mm defect and repaired with the nerve conduits. All of the 32 Wistar rats were randomly divided into 4 groups: group PLGL-RGD-NGF, group PLGL-RGD, group PLGL and group autograft. At 3 months after surgery, the regenerated rat sciatic nerve was evaluated by footprint analysis, electrophysiology, and histologic assessment. Experimental data were processed using the statistical software SPSS 10.0.

**Results:**

The sciatic function index value of groups PLGL-RGD-NGF and autograft was significantly higher than those of groups PLGL-RGD and PLGL. The nerve conduction velocities of groups PLGL-RGD-NGF and autograft were significantly faster than those of groups PLGL-RGD and PLGL. The regenerated nerves of groups PLGL-RGD-NGF and autograft were more mature than those of groups PLGL-RGD and PLGL. There was no significant difference between groups PLGL-RGD-NGF and autograft.

**Conclusions:**

PLGL-RGD-NGF nerve conduits are more effective in regenerating nerves than both PLGL-RGD nerve conduits and PLGL nerve conduits. The effect is as good as that of an autograft. This work established the platform for further development of the use of PLGL-RGD-NGF nerve conduits for clinical nerve repair.

## Background

Peripheral nerve injuries are frequently caused by trauma and may lead to a significant loss of sensory or motor functions. Numerous surgeries have been carried out each year for nerve injury repairing. For short nerve injuries,direct end-to-end suturing techniques are suggested. For severe nerve injuries, autologous nerve graft has been the first choice. However, autologous nerve graft is limited by the availability of expendable donor nerves and donor site morbidity [1,2]. Repairing alternative approach is to develop synthetic nerve conduits to bridge the gaps between the proximal and the distal nerve stumps for promoting nerve regeneration. Studies over the past few decades have resulted in several clinically available nerve conduits, such as Neurotube (polyglycolic acid, PGA, Synovis), Neurolac (poly-DL-lactide-caprolactone, PLCL, Polyganics BV), NeuraGen (collagen type I, Integra NeuroSciences) and Neuro-Matrix/Neuroflex (collagen type I, Collagen Matrix Inc) [3-11]. NeuraGen and Neuro-Matrix/Neuroflex were fabricated out of collagen with favorable results in nerve repair [12,13], but they are rather expensive and difficult to handle during suturing, furthermore, they only can bridge short defects because of their mechanical weakness [14]. Because of their good biodegradability, biocompatibility and mechanical properties, Neurotube and Neurolac were approved by U.S. Food and Drug Administration (FDA) and Conformit Europe (CE) for clinical nerve repair [1]. Nevertheless, current research indicates that inert nerve conduits such as Neurotube and Neurolac cannot sustain optimal axonal growth, and specific modifications are required to permit successful nerve regeneration and functional recovery [15]. It is believed that an ideal nerve conduit should provide not only structural support for damaged nerves but also the neurotropic and neurotrophic support for axonal regrowth. Numerous experiments indicated that, after nerve injury, cell adhesion molecules and neurotrophic factors played very important roles in nerve regeneration and functional recovery [16-24].

Two major extracellular matrix (ECM) constituents, laminin and fibronectin, are thought to promote cell adhesion. However, immune reactivity and protein denaturation are concerned. To overcome the problem, the adhesive peptides, containing Arg-Gly-Asp (RGD) sequence, have been tested as alternatives to native ECM proteins in the past decades [25]. Adsorption of RGD peptides to polyesters surface was usually attempted, but the retention time of the RGD peptides was too short for practical application. Chemical modification might be an alternative approach, however, polyesters such as PGA and PLCL lack functional groups to covalently bonding RGD peptides. We have previously synthesized poly{(lactic acid)-*co*-[(glycolic acid)-*alt*-(L-lysine)]} (PLGL), which incorporates free amines and can covalently bonding RGD peptides [26].

Nerve growth factor (NGF) is the most thoroughly characterized neurotrophic factor. NGF can ensure the survival of the cell bodies and support the regeneration of the axons toward specific target organs. For improvement of nerve conduits, a large number of studies have focused on the addition of NGF within the conduits [18-24]. Xu X et al. reported the preparation of NGF-encapsulated microspheres for the delivery of NGF. However, the organic solvents had unfavorable effects on the native structure of NGF, resulting in the deactivation of NGF during the encapsulation procedure [27]. Chen P R et al. reported immobilizing NGF on gluataraldehyde cross-linking gelatin membranes with carbodiimide for axonal regeneration. The NGF immobilized on the membrane still kept bioactivity that could promote the neurite outgrowth of pheochromocytoma (PC12) cell, but the gelatin membrane turned to more hydrophobic surface after cross-linked by aldehyde group. This hydrophobic surface of the membranes resulted in poor cell adhesion [28].

Since the carboxyl groups of PLGL-RGD molecules can also react with the amine groups of NGF molecules in the presence of carbodiimide, we combined PLGL-RGD with NGF to construct a double-functional PLGL-RGD-NGF nerve conduits. The purpose of this study was to investigate the efficiency of PLGL-RGD-NGF nerve conduits to enhance nerve regeneration and functional recovery through the rat sciatic nerve transection model.

## Methods

### Materials

PLGL (Mw = 1.5 × 10^5^) was synthesized in our laboratory. Gly-Arg-Gly-Asp-Gly (RGD Peptide, GL Biochem), 1,ĺ-carbonyldiimidazole (CDI, GL Biochem), nerve growth factor (NGF, Sigma, 7S NGF), 1-ethyl-3-(3-dimethylaminopropyl) carbodiimide (EDAC, Aldrich), other reagents were all of analytical grade. Wistar rats were purchased from Tongji Medicinal School, Huazhong University of Technology.

#### RGD peptide modification of PLGL

PLGL (2.5g) was dissolved in 50 ml of dichloromethane. RGD Peptide (50 mg) was dissolved in 20 ml of dimethyl sulfoxide and added to the resulting solution. CDI (42.5 mg) dissolved in 1 ml of dichloromethane was added to the mixture solution. The reaction mixture was stirred for 4 h. After removal of dichloromethane in vacuum, methanol was added to complete copolymer precipitation. The precipitate was dried under high vacuum.

#### NGF modification of PLGL-RGD membranes

PLGL-RGD was dissolved in ethyl acetate at a concentration of 10wt%. PLGL-RGD membranes were prepared using the solvent evaporation method. PLGL-RGD membranes were soaked in 20 mL EDAC solution with a concentration of 1mg/mL for 24h at 4°C. Unbound and excess EDAC was removed by washing the membranes with double-distilled water. The membranes were then transferred to 10 mL NGF solution with different concentration of 10, 25, 40 and 50ng/mL for 24 h at 4°C. The unbound NGF was removed by washing the membranes with the phosphate buffer solution (PBS, pH=7.4). After NGF was immobilized on PLGL-RGD membranes, the soaking solution and the phosphate buffer solution were collected and the amout of NGF of these solution was measured by ELISA. The amount of NGF immobilized on the PLGL-RGD membrane could be calculated by that the total NGF in the soaking solution for immobilization subtracted by the amount of uncross-linked NGF.

#### Preparation of PLGL-RGD–NGF, PLGL-RGD and PLGL nerve conduits

The side containing NGF of PLGL-RGD–NGF membranes were rolled in an inside of a conduit, while the opposite side of them were sealed to fabricate the PLGL-RGD–NGF conduits. PLGL membranes were prepared using the solvent evaporation method. PLGL and PLGL-RGD membranes were rolled to fabricate PLGL and PLGL-RGD conduits. The final length of nerve conduits was 14 mm, with an inner diameter of 2.0 mm and a tube wall thickness of 0.2 mm. All membranes or nerve conduits were sterilized with ultraviolet light for the later experiment.

#### Cell culture

Schwann cells from neonatal Wistar rats were isolated and expanded in culture using methods described by Honkanen H et al. [29].

Schwann cells were seeded on PLGL-RGD–NGF,PLGL-RGD and PLGL membranes at a density of 1 × 10^6^ cells/cm^2^, incubated in a humidified incubator at 37°C and 5% CO_2_. On day 5, the membranes with cells attached were washed three times with PBS and then fixed with 3.7% formaldehyde for 30 min, and washed again with PBS. The cells were incubated with FITC-phalloidin (5 ug/mL, Sigma, Dorset) for 1 h. After washed three times with PBS, the cells were stained with Hoechst 33258 for 10 min and photographed using a laser confocal microscope.

Cell viability on the membranes was characterized by MTT assay. PLGL-RGD–NGF,PLGL-RGD and PLGL membranes were cut into small circular pieces with a diameter of 6.38 mm and placed in a 96-well culture plate. Schwann cells were seeded in 96 well culture plates at a density of 1 × 10^5^ cells/well, incubated in a humidified incubator at 37°C and 5% CO_2_. After culturing for 1 day, 3 days and 5 days respectively, the supernatant was removed. 20 μL MTT solution (5 mg/mL) was added to each culture well and incubated for 4 h. The absorbance at 490 nm was recorded by an automatic enzyme scanner. Five parallels for every sample were averaged.

#### Animals and surgical procedure

All procedures undertaken in this study were approved by the Animal Care and Use Committees of Wuhan university of technology and conformed to NIH guidelines.

Thirty-two male Wistar rats were randomized into 4 groups, 8 weeks old at the beginning of the experiments, 8 rats each: group PLGL-RGD-NGF, group PLGL-RGD, group PLGL and group autograft. The rats were anesthetized with 40mg/kg pentobarbital sodium. The right sciatic nerve was exposed after skin incision, and separation of muscles around the nerve tissues using blunt dissection. Subsequently, the right sciatic nerve was severed into proximal and distal segments in the middle of the right thigh. Defects of 10 mm in the sciatic nerve were created by surgical removal of the nerve tissue. Both the proximal and the distal stumps were secured with 9-0 nylon to a depth of 2 mm into the conduits, leaving a 10 mm gap between the stumps in groups PLGL-RGD-NGF, PLGL-RGD and PLGL. In group autograft, the nerve defect was bridged with the resected nerve segment, which was reversed and anastomosed to the proximal and distal nerve stumps. The wound was closed in two layers with 6-0 nylon sutures.

#### Footprint analysis

After dipping the hind feet in carbon ink, the rat was placed in a paper-lined walkway (8 × 42 cm) that led into a darkened cage [30]. Footprints left on the paper as the rat walked down the track.

The footprints were measured according to three parameters: (i) distance from the heel to the third toe, the print length (PL), (ii) distance from the first to the fifth toe, the toe spread (TS), and (iii) distance from the second to the fourth toe, the intermediate toe spread (ITS). The data were used to compute: (i) print length factor (PLF) = (EPL-NPL)/NPL, (ii) toe spread factor (TSF) = (ETS-NTS)/NTS, and (iii) intermediary toe spread factor (ITSF) = (EITS-NITS)/NITS, where E is for the experimental side and N is for the normal control. These factors were adapted to the Bain-Mackinnon-Hunter sciatic function index (SFI) [31] by the formula,

(1) SFI=−38.3×PLF+109.5×TSF+13.3×ITSF−8.8

#### Electrophysiologic assessment

At 3 months after surgery, electrophysiologic tests were performed on all animals. Animals were anesthetized with sodium pentobarbital, the previous surgical site was reopened, and the sciatic nerve was re-exposed. Single electrical pulses were delivered via bipolar electrodes placed in turn at the proximal and distal trunk of the grafted nerve. Compound muscle action potentials (CMAPs) were recorded via an electrode inserted into the anterior tibial muscle. The difference in latency of CMAPs was computed, and the distance between the proximal and distal sites of stimulation was measured for the determination of the conduction velocity across the regenerated nerve.

#### Histological evaluation

The implanting conduits were harvested immediately after the electrophysiologic tests. The nerve specimens were fixed in 4% glutaraldehyde for 24 h. The regenerated nerve stumps in the proximal and distal directions were removed. The 5mm middle segments of the regenerated nerves in each group were embedded with olefin and then cut into 4-μm thickness for H and E staining and S-100 immunohistochemistry analysis. Other 5mm middle segments of the regenerated nerves in each group were embedded with epoxy resin. The thin–thick slices of 1.0 μm were made and stained with methylene blue. All the nerve sections were observed under a light microscope. To minimize the error, at 400 × magnification, six measurement windows were randomly selected by an observer blinded to the experiment to count each nerve, using HPIAS-1000 high-resolution color graphic report of the pathology analysis system to analyze the photographs for the determination of the myelinated axon density, myelin thickness, and the diameter of axons of the regenerated nerve. Analysis of the digitalized information, based on gray and white scales, was performed by the computer software. Axons were identified by the deeply stained color. A total area of myelinated fibers was measured for each nerve specimen. Thickness of axon sheaths on cross section was also measured by the morphometric computer software. The ultrathin slices of 50 nm were made and stained with lead citrate and uranylacetate, and then were examined under a transmission electron microscope (Tecnai, FEI).

#### Statistical analysis

Experimental data were processed using the statistical software SPSS 10.0 (Bizinsight, Beijing China), and expressed as mean ± standard error of mean. Statistical differences were analyzed using single-factor analysis. The means of the two groups were compared using least significant difference law (*α* = 0.05).

## Results

### RGD peptide modification

It was reported that peptides could be attached to poly(lactic acid-*co*-lysine) using CDI as a linking agent [32]. In the present work, we used the same method to couple RGD peptide to PLGL. Amino acid analysis (AAA) demonstrated that RGD peptide was coupled to the copolymer at a concentration of 9.2-17.3 μmol/g.

### NGF immobilization

**Figure 1:**
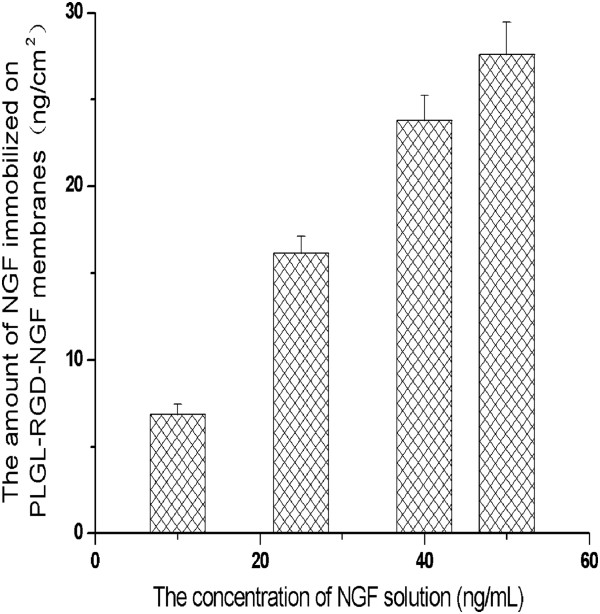
The amount of NGF immobilized on PLGL-RGD-NGF membranes (n = 5).

In the study, the NGF solution of different concentration was used to immobilize NGF molecules on PLGL-RGD membranes. As shown in Figure 1, the number of NGF immobilized on PLGL-RGD membranes was increasing from 6.85 ng/cm^2^ to 27.6 ng/cm^2^ with the NGF concentration of the soaking solution.

### Cell culture

**Figure 2:**
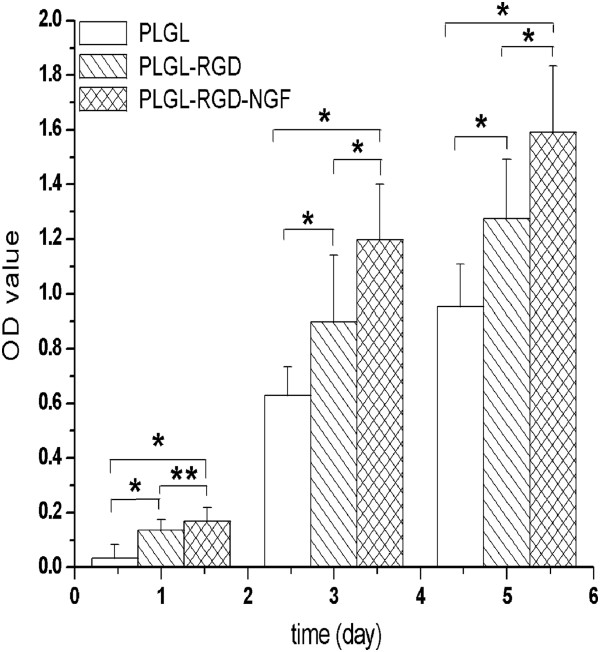
The viability of schwann cells seeded on PLGL, PLGL-RGD and PLGL-RGD-NGF membranes assayed by MTT on days 1, 3 and 5 respectively (*p<0.05, **p > 0.05).

**Figure 3:**
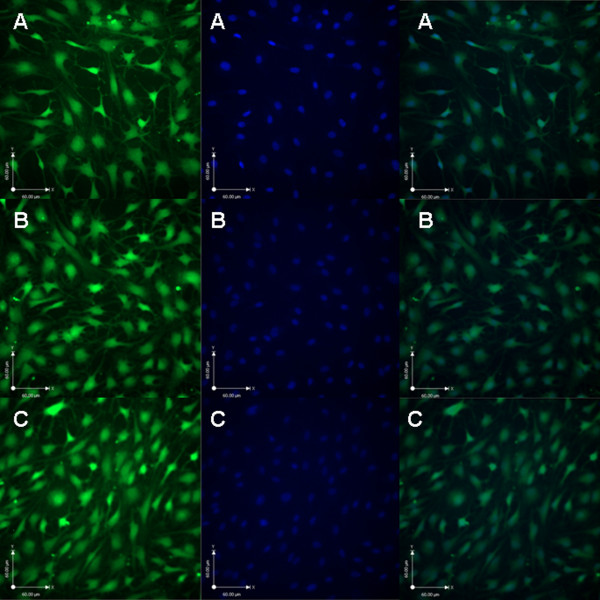
**Schwann cells morphology cultured on the different membranes for 5 days.A**: PLGL membrane, **B**: PLGL-RGD membrane, **C**: PLGL-RGD-NGF membrane.

As shown in Figure 2, the OD value of each test group increased with the cultured time. At the first day, the OD values of group PLGL-RGD-NGF were similar to group PLGL-RGD (*P* > 0.05). From day 3 to day 5, the OD value of group PLGL-RGD-NGF was higher than that of group PLGL-RGD (P < 0.05). From day 1 to day 5, the OD values of groups PLGL-RGD-NGF and PLGL-RGD were higher than that of group PLGL (P < 0.05). As shown in Figure 3, Schwann cells on PLGL-RGD-NGF and PLGL-RGD membranes also adhered and extended better than those on PLGL membranes. Schwann cells on PLGL-RGD-NGF membrane appeared with a characteristic spindle shape.

### Footprint analysis

**Figure 4:**
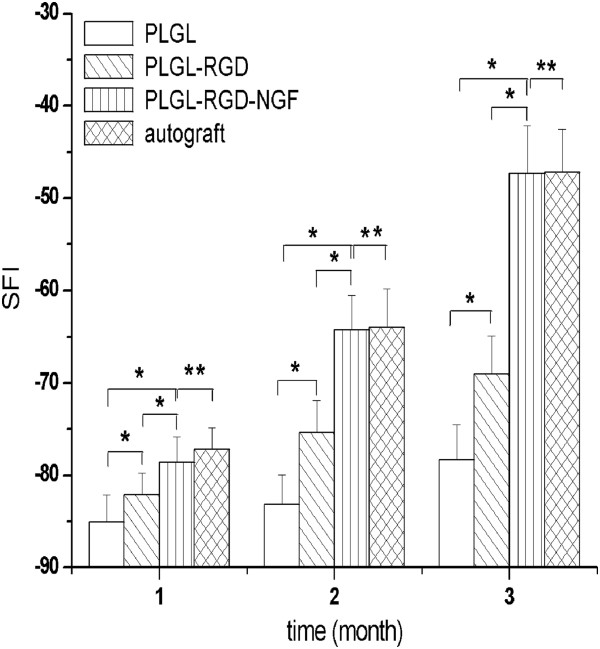
Sciatic function indices of all animal groups (*p<0.05, **p > 0.05).

**Figure 5:**
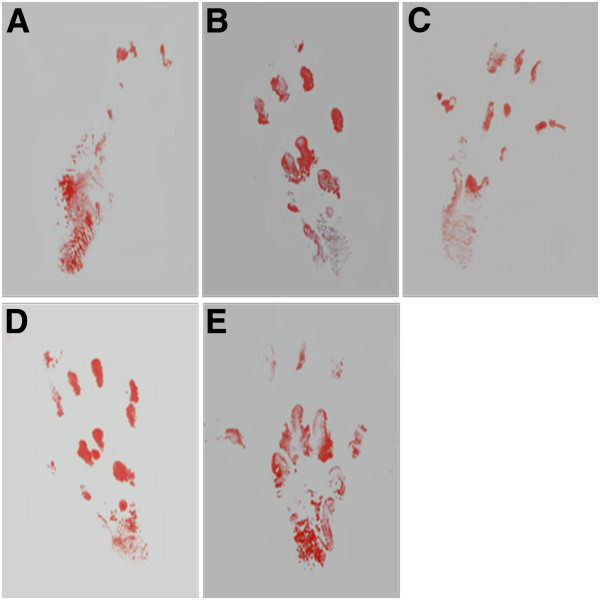
**The rats’ walking track of different groups at 3 months after surgery.A**: group PLGL, **B**: group PLGL-RGD, **C**: group PLGL-RGD-NGF, **D**: group autograft, **E**: normal group.

The SFI value was calculated in only 26 rats because of automutilations and paw contractions. There was no significant difference between the four groups at the beginning 1 month. But 1 month later, the SFI value of groups PLGL-RGD-NGF and autograft was significantly higher than the other groups (p < 0.05). The SFI value of group PLGL-RGD-NGF was similar to that of group autograft (p > 0.05, Figures 4 and 5).

### Electrophysiologic assessment

**Figure 6:**
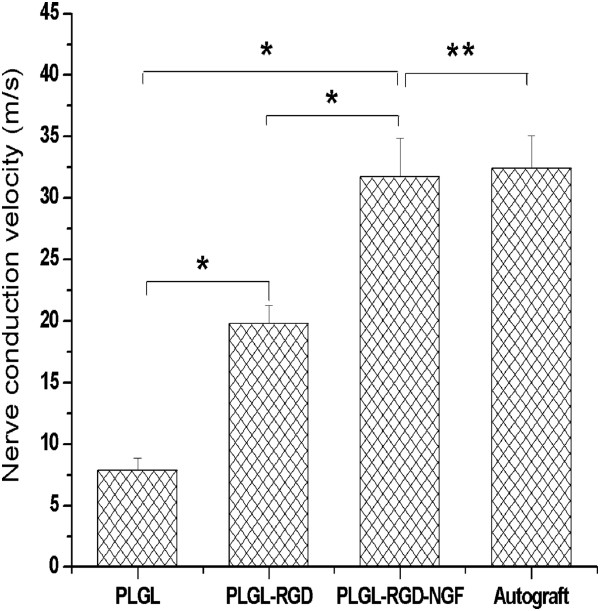
Nerve conduction velocities in groups PLGL, PLGL-RGD, PLGL-RGD-NGF and autograft (*p<0.05, **p > 0.05).

At 3 months after surgery, CMAPs were detected in all groups on the side bearing the bridged sciatic nerve. Based on latencies of the CMAPs, nerve conduction velocities (NCVs) were determined (Figure 6). The NCVs of group PLGL-RGD-NGF were significantly faster than those of groups PLGL-RGD and PLGL (P < 0.05). The NCVs of group PLGL-RGD-NGF was similar to those of group autograft (P > 0.05).

### Histological assessment

**Figure 7:**
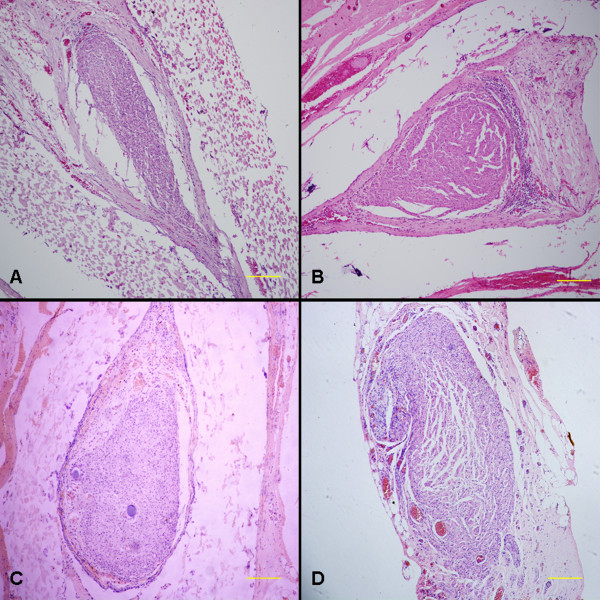
**Hematoxylin/eosin staining of the regenerated nerves in different groups at 3 months after surgery.** Scale Bar: 200μm. **A**: group PLGL, **B**: group PLGL-RGD, **C**: group PLGL-RGD-NGF, **D**: group autograft.

**Figure 8:**
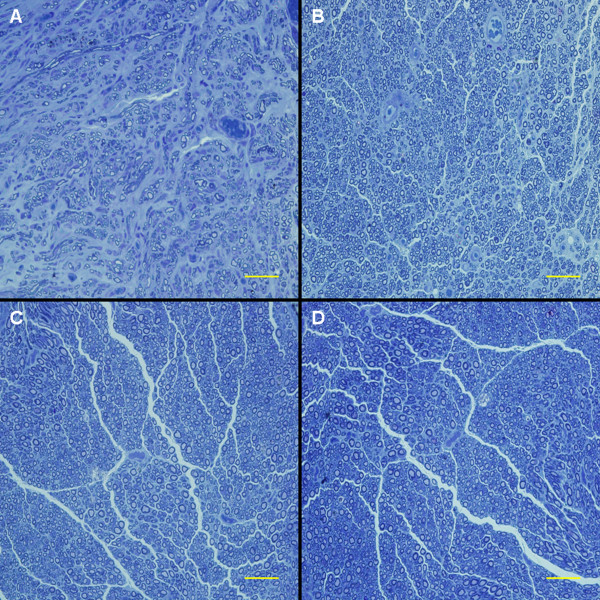
**Regenerated nerve stained with methylene blue in different groups at 3 months after surgery.** Scale Bar: 50μm. **A**: group PLGL, **B**: group PLGL-RGD, **C**: group PLGL-RGD-NGF, **D**: group autograft.

**Figure 9:**
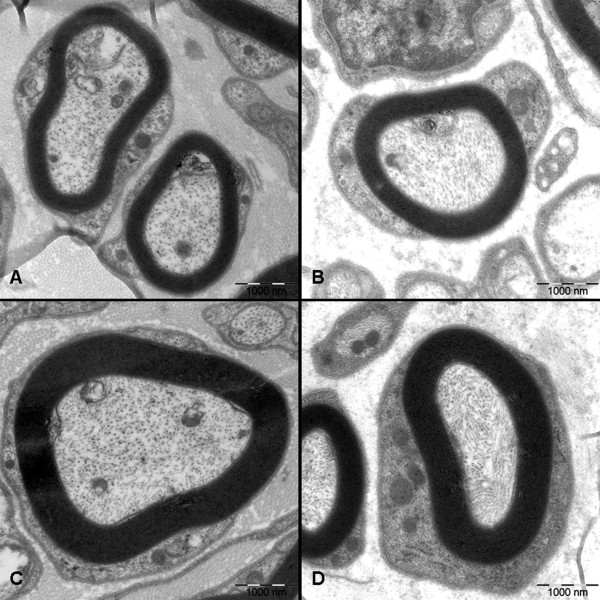
**Transmission electron microscope images of the regenerated nerve in different groups at 3 months after surgery.A**: group PLGL, **B**: group PLGL-RGD, **C**: group PLGL-RGD-NGF, **D**: group autograft.

**Figure 10:**
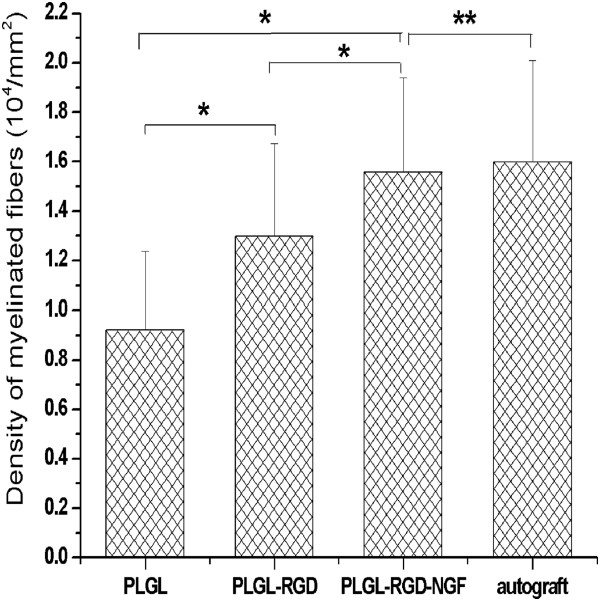
The density of myelinated fibers in groups PLGL, PLGL-RGD, PLGL-RGD-NGF and autograft (*p<0.05, **p > 0.05).

**Figure 11:**
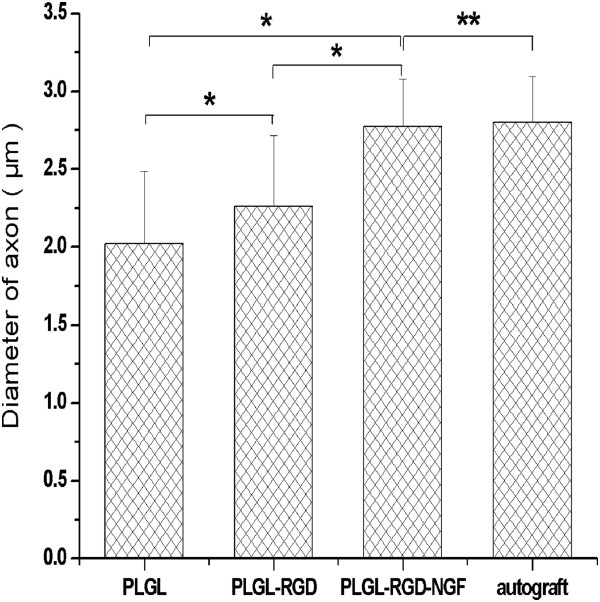
The mean diameter of axon in groups PLGL, PLGL-RGD, PLGL-RGD-NGF and autograft (*p<0.05, **p > 0.05).

**Figure 12:**
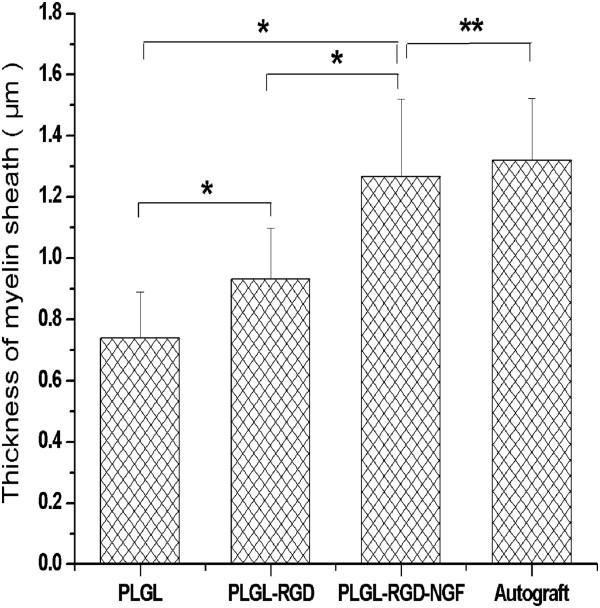
The average thickness of myelin sheath in groups PLGL, PLGL-RGD, PLGL-RGD-NGF and autograft (*p<0.05, **p > 0.05).

**Figure 13:**
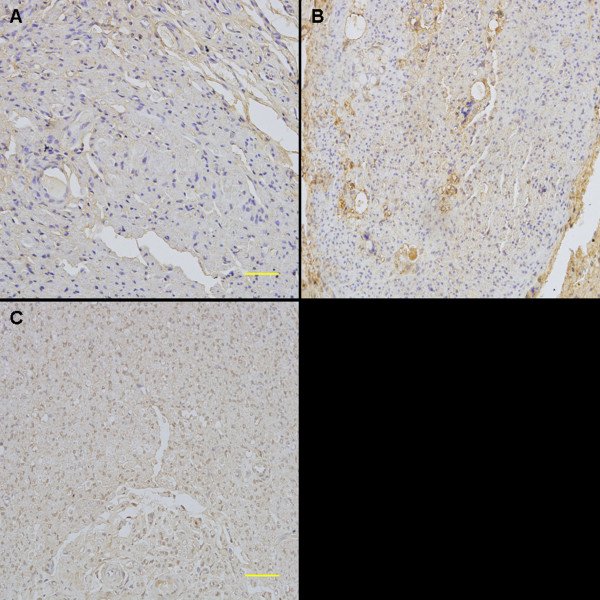
**The immunocytochemical staining of S-100 of the regenerated nerves in different groups at 3 months after surgery. Scale Bar: 50μm.A**: group PLGL, **B**: group PLGL-RGD, **C**: group PLGL-RGD-NGF.

**Figure 14:**
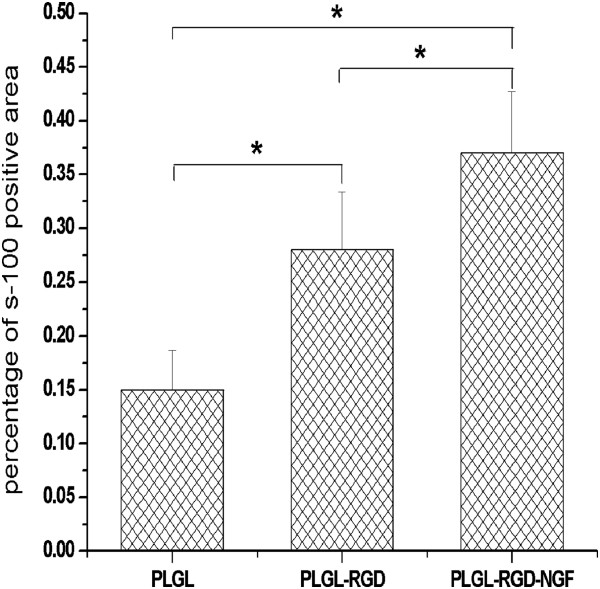
The statistical analysis of S-100 positive area of the regenerated nerve in groups PLGL, PLGL-RGD and PLGL-RGD-NGF (*p<0.05).

At 3 months after surgery, the regenerated nerve had grown cross through the 10 mm gap. H and E staining (Figure 7), methylene blue staining (Figure 8), and lead citrate and uranylacetate staining (Figure 9) were performed to investigate the newborn nerves of each group. For group PLGL-RGD-NGF, the regenerated nerve fibers dispersed densely and the myelinated fibers had a compact and uniform structure including clear electron-dense myelin sheath and perfect basal membrane of Schwann cells. In addition, statistical analysis revealed that there was no significant difference in the morphometric data, including the density of myelinated fibers, the mean diameter of myelinated fibers and the average thickness of myelin sheath, between groups PLGL-RGD-NGF and autograft (p > 0.05). The density of myelinated fibers, the mean diameter of axon and the average thickness of myelin sheath in group PLGL-RGD-NGF were significantly higher than those in groups PLGL-RGD and PLGL (p < 0.05) (Figures 10, 11 and 12). Consistent with the the result of cell culture, the S-100 positive expression of group PLGL-RGD-NGF was the strongest compared with groups PLGL-RGD and PLGL (p < 0.05). The S-100 positive expression of group PLGL-RGD was stronger than that of group PLGL (p < 0.05) (Figures 13 and 14).

## Discussion

Transection is one of the most severe injuries in the peripheral nerve. Although peripheral nerves have the potential for regenerating after injury, this capacity is strictly depending on the microenvironment for regeneration. At present there are a few synthetic nerve conduits approved by FDA and CE for clinical nerve repair. These are rudimental conduits that act as a guide for the regenerating axons and as a barrier against the ingrowth of scar-forming tissue. However physical nerve guidance by them may not be sufficient to foster optimal nerve regeneration and functional recovery. NGF is also required in the treatment of peripheral nerve injury to ensure the survival of the cell bodies and to support the regeneration of the axons toward specific target organs. However NGF supplementation alone is insufficient for substantive axonal regrowth. At the same time, cell adhesion molecules such as RGD peptide also play an indispensable role in supporting axonal regrowth. Therefore, we modified PLGL with RGD peptide and NGF to fabricate new PLGL-RGD-NGF nerve conduits.

A very small concentration of RGD peptides was shown to have enhanced biological effects, for instance, nonadherent surface on incorporation of 1 fmol/cm^2^ of an RGD peptide concentration effectively promoted cell adhesion [32]. In this study, we prepared PLGL-RGD membranes containing 9.2-17.3 μmol/g RGD peptide. Cell culture showed that Schwann cells on PLGL-RGD-NGF and PLGL-RGD membranes exhibited significantly higher total viability than those on PLGL membranes from day 1 to day 5 (P < 0.05) (Figure 2). These demonstrated that RGD peptide of PLGL-RGD-NGF and PLGL-RGD membranes could effectively enhance Schwann cells adhesion. Interestingly, from day 3 to day 5, Schwann cells on PLGL-RGD-NGF membranes also exhibited higher viability than those on PLGL-RGD membranes (Figure 2). This could be due to cell adhesion molecule expression on Schwann cells which can be directly regulated by NGF of PLGL-RGD-NGF membranes [33].

NGF has been shown to enhance neuron survival and stimulate nerve regeneration. However, NGF has a short half-life in the body. In order to prolong the availability of NGF to targeted tissues, we immobilized NGF on the nerve conduits with EDAC. The linking between NGF and PLGL-RGD membranes was through the carboxyl groups on the surface of PLGL-RGD membranes reacting with the amine groups of NGF in the presence of EDAC. The amount of NGF immobilized on PLGL-RGD membranes was concerned with not only the NGF concentration of the soaking solution but also the number of the carboxyl groups on the surface of PLGL-RGD membranes. Because Bhang SH et al. reported that 100 ng/mL immobilized NGF could effectively enhance cell viability, mitochondrial metabolic activity, and neuronal differentiation. Moreover, cell viability, mitochondrial metabolic activity, and neuronal differentiation signaling due to immobilized NGF could be sustained for longer periods than by NGF added to culture medium [34]. In the present study, we only choosed 10ng/mL NGF to modify PLGL-RGD membranes. There was about 6.85 ng/cm^2^ of NGF grafted on the PLGL-RGD membranes. In this context, PLGL-RGD-NGF nerve conduits (2 mm diameter × 14 mm length) contained about 137 ng/mL NGF. If we choosed 50 ng/mL NGF to modify PLGL-RGD membranes. There was about 27.6 ng/cm^2^ of NGF grafted on the PLGL-RGD membranes. PLGL-RGD-NGF nerve conduits (10mm diameter) also contained about 110.4 ng/mL NGF. Therefore, by selecting different NGF concentration, PLGL-RGD-NGF nerve conduits of different sizes should contain enough NGF to promote neural cell growth and differentiation.

SFI is a simple and repeatable method for evaluating the functional condition of sciatic nerve injuries. A straightforward measure of muscle innervation is the amplitude of CMAPs following electrical stimulation of the nerve. The muscles to be assessed can be defined, and the technique is easily applicable to rats [35]. But considerable variations in depth of insertion of the recording electrode in muscles were observed even with supramaximal stimulation intensities. Therefore, in this study, the amplitude of CMAPs was not used for assessment of the functional recovery of the sciatic nerves. Rather, the latency for eliciting CMAPs was used for the determination of conduction velocity along the bridged nerve. After transection the nerve conduction pathway had been completely destroyed. The SFI value dramatically decreased and NCV could not be detected at the first month after the operation. At 3 months after surgery, the SFI value and NCV of groups PLGL-RGD-NGF and autograft were significantly higher than those of groups PLGL-RGD and PLGL. The SFI value and NCV of group PLGL-RGD-NGF were similar to that of group autograft. These showed that functional recovery of groups PLGL-RGD-NGF and autograft were quicker and superior than that of groups PLGL-RGD and PLGL.

In S-100 immunocytochemical analysis, we could observed stronger S-100 positive expression in group PLGL-RGD-NGF than that of groups PLGL-RGD and PLGL. Histological assessment showed that the density of myelinated fibers, the mean diameter of axon and the average thickness of myelin sheath in group PLGL-RGD-NGF were similar to those of group autograft, but significantly higher than those of groups PLGL-RGD and PLGL. These showed that PLGL-RGD-NGF nerve conduits could effectively enhance Schwann cells adhesion and migration, axonal growth and the maturity of regenerated myelin sheaths.

## Conclusion

New PLGL-RGD-NGF nerve conduits were fabricated by grafting RGD peptides and NGF on PLGL to promote nerve regeneration and functional recovery. The results of cell culture showed that PLGL-RGD-NGF more efficiently enhanced Schwann cells adherence and growth than PLGL. For a 10mm defect in the rat sciatic nerve, nerve regeneration and functional recovery of PLGL-RGD-NGF nerve conduits performed similar to autograft and better than PLGL nerve conduits. This work established the platform for further development of the use of PLGL-RGD-NGF nerve conduits for clinical nerve repair.

## Abbreviations

PLGL, Poly{(lactic acid)-*co*-[(glycolic acid)-*alt*-(L-lysine)]}; RGD peptide, Gly-Arg-Gly-Asp-Gly; NGF, Nerve growth factor; PGA, Polyglycolic acid; PLCL, Poly-DL-lactide-caprolactone; FDA, Food and drug administration; CE, Conformit Europe; ECM, Extracellular matrix; PC12 cell, Pheochromocytoma cell; EDAC, 1-ethyl-3-(3-dimethylaminopropyl) carbodiimide; PBS, Phosphate buffer solution; SFI, Sciatic function index; CMAPs, Compound muscle action potentials; NCVs, Nerve conduction velocities.

## Competing interests

The authors declare that they have no competing interests.

## Authors' contributions

QY performed experiments and analysis and wrote the paper. YY performed experiments and analysis. BL carried out cell culture studies. All authors read and approved the final manuscript.

## References

[B1] Jiang X Lim SH Mao HQ Chew SY Current applications and future perspectives of artificial nerve conduits Exp Neurol 2010 223 86 101 10.1016/j.expneurol.2009.09.009 19769967

[B2] Lundborg G A 25-year perspective of peripheral nerve surgery: Evolving neuroscientific concepts and clinical significance J Hand Surg Am 2000 25 391 394 10.1053/jhsu.2000.4165 10811744

[B3] Bell JHA Haycock JW Next generation nerve guides: materials, fabrication, growth factors, and cell delivery Tissue Eng Part B Rev 2012 18 116 128 10.1089/ten.teb.2011.0498 22010760

[B4] Huang W Begum R Barber T Ibba V Tee NCH Arastoo M Yang Q Robson LG Lesage S Gheysens T Skaer NJV Knight DP Priestley JV Regenerative potential of silk conduits in repair of peripheral nerve injury in adult rats Biomaterials 2012 33 59 71 10.1016/j.biomaterials.2011.09.030 22005069

[B5] McGrath AM Brohlin M Kingham PJ Novikov LN Wiberg M Novikova LN Fibrin conduit supplemented with human mesenchymal stem cells and immunosuppressive treatment enhances regeneration after peripheral nerve injury Neurosci Lett 2012 516 171 176 10.1016/j.neulet.2012.03.041 22465323

[B6] Ignatiadis IA Yiannakopoulos CK Barbitsioti AD Avram AM Patralexis HG Tsolakis CK Papalois AE Xenakis TH Beris AE Soucacos PN Diverse types of epineural conduits for bridging short nerve defects: An experimental study in the rabbit Microsurgery 2007 27 98 104 10.1002/micr.20313 17290376

[B7] Wang XD Hu W Cao Y Yao J Wu J Gu XS Dog sciatic nerve regeneration across a 30-mm defect bridged by a chitosan/PGA artificial nerve graft Brain 2005 128 1897 1910 10.1093/brain/awh517 15872018

[B8] Nakayama K Takakuda K Koyama Y Itoh S Wang W Mukai T Shirahama N Enhancement of peripheral nerve regeneration using bioabsorbable polymer tubes packed with fibrin gel Artif Organs 2007 31 500 508 10.1111/j.1525-1594.2007.00418.x 17584474

[B9] Schnell E Klinkhammer K Balzer S Brook G Klee D Dalton P Mey J Guidance of glial cell migration and axonal growth on electrospun nanofibers of polyepsilon-caprolactone and a collagen/poly-epsiloncaprolactone blend Biomaterials 2007 28 3012 3025 10.1016/j.biomaterials.2007.03.009 17408736

[B10] Alluin O Wittmann C Marqueste T Chabas JF Garcia S Lavaut MN Guinard D Feron F Decherchi P Functional recovery after peripheral nerve injury and implantation of a collagen guide Biomaterials 2009 30 363 373 10.1016/j.biomaterials.2008.09.043 18929405

[B11] Ao Q Fung CK Tsui AYP Cai S Zuo HC Chan YS Shum DKY The regeneration of transected sciatic nerves of adult rats using chitosan nerve conduits seeded with bone marrow stromal cell-derived Schwann cells Biomaterials 2011 32 787 796 10.1016/j.biomaterials.2010.09.046 20950852

[B12] Archibald SJ Shefner J Krarup C Madison RD Monkey median nerve repaired by nerve graft or collagen nerve guide tube J Neurosci 1995 15 4109 4123 7751969 10.1523/JNEUROSCI.15-05-04109.1995 PMC6578227

[B13] Itoh S Takakuda K Kawabata S Aso Y Kasai K Itoh H Shinomiya K Evaluation of cross-linking procedures of collagen tubes used in peripheral nerve repair Biomaterials 2002 23 4475 4481 10.1016/S0142-9612(02)00166-7 12322967

[B14] Lorenz U Marta M Michael P Hans PM Bruno G Lorenz M Silk fibroin matrices for the controlled release of nerve growth factor (NGF) Biomaterials 2007 28 4449 4460 10.1016/j.biomaterials.2007.06.034 17643485

[B15] Moore AM Kasukurthi R Farhadi HF Borschel GH Limitations of Conduits in Peripheral Nerve Repairs Hand 2009 4 180 186 10.1007/s11552-008-9158-3 19137378 PMC2686795

[B16] Webber C Zochodne D The nerve regenerative microenvironment: Early behavior and partnership of axons and Schwann cells Exp Neurol 2010 223 51 59 10.1016/j.expneurol.2009.05.037 19501085

[B17] Madduri S Papaloϊzos M Gander B Trophically and topographically functionalized silk fibroin nerve conduits for guided peripheral nerve regeneration Biomaterials 2010 31 2323 2334 10.1016/j.biomaterials.2009.11.073 20004018

[B18] Fine EG Decosterd I Papaloizos M Zurn AD Aebischer P GDNF and NGF released by synthetic guidance channels support sciatic nerve regeneration across a long gap Eur J Neurosci 2002 15 589 601 10.1046/j.1460-9568.2002.01892.x 11886440

[B19] Lee AC Yu VM Lowe JB Brenner MJ Hunter DA Mackinnon SE Sakiyama-Elbert SE Controlled release of nerve growth factor enhances sciatic nerve regeneration Exp Neurol 2003 184 295 303 10.1016/S0014-4886(03)00258-9 14637100

[B20] Burdick JA Ward M Liang E Young MJ Langer R Stimulation of neurite outgrowth by neurotrophins delivered from degradable hydrogels Biomaterials 2006 27 452 459 10.1016/j.biomaterials.2005.06.034 16115674

[B21] Dodla MC Bellamkonda RV Differences between the effect of anisotropic and isotropic laminin and nerve growth factor presenting scaffolds on nerve regeneration across long peripheral nerve gaps Biomaterials 2008 29 33 46 10.1016/j.biomaterials.2007.08.045 17931702 PMC2682535

[B22] Pfister LA Alther E Papaloïzos M Merkle HP Gander B Controlled nerve growth factor release from multi-plyalginate/chitosan-based nerve conduits Eur J Pharm Biopharm 2008 69 563 572 10.1016/j.ejpb.2008.01.014 18294826

[B23] Wood MD Moore AM Hunter DA Tuffaha S Borschel GH Mackinnon SE Sakiyama-Elbert SE Affinity-based release of glial-derived neurotrophic factor from fibrin matrices enhances sciatic nerve regeneration Acta Biomater 2009 5 959 968 10.1016/j.actbio.2008.11.008 19103514 PMC2678870

[B24] Chung TW Yang MC Tseng CC Sheu SH Wang SS Huang YY Chen SD Promoting regeneration of peripheral nerves in-vivo using new PCL-NGF/Tirofiban nerve conduits Biomaterials 2011 32 734 743 10.1016/j.biomaterials.2010.09.023 20888633

[B25] Bellis SL Advantages of RGD peptides for directing cell association with biomaterials Biomaterials 2011 32 4205 4210 10.1016/j.biomaterials.2011.02.029 21515168 PMC3091033

[B26] Yan QJ Li J Li SP Yin YX Zhang P Synthesis and RGD peptide modification of poly{(lactic acid)-co-[(glycolic acid)-alt-(L-lysine)]} e-Polymers 2008 28 1 12

[B27] Xu X Yu H Gao S Mao H-Q Leong KW Wang S Polyphosphoester microspheres for sustained release of biologically active nerve growth factor Biomaterials 2002 23 3765 3772 10.1016/S0142-9612(02)00116-3 12109702

[B28] Chen PR Chen MH Lin FH Su WY Release characteristics and bioactivity of gelatin-tricalcium phosphate membranes covalently immobilized with nerve growth factors Biomaterials 2005 26 6579 6587 10.1016/j.biomaterials.2005.03.037 16023717

[B29] Henrika Honkanen Outi Lahti Marja Nissinen Myllyla RM Kangas S Paivalainen S Alanne MH Peltonen S Peltonen J Heape AM Isolation, purification and expansion of myelination-competent, neonatal mouse Schwann cells Eur J Neurosci 2007 26 953 964 10.1111/j.1460-9568.2007.05726.x 17714189

[B30] Meek MF Den Dunnen WFA Schakenraad JM Robinson PH Long-term evaluation of functional nerve recovery after reconstruction with a thinwalled biodegradable poly (DL-lactide-epsilon-caprolactone) nerve guide, using walking track analysis and electrostimulation tests Microsurgery 1999 19 247 253 10.1002/(SICI)1098-2752(1999)19:5<247::AID-MICR7>3.0.CO;2-E 10413791

[B31] Bain JR Mackinnon SE Hunter DA Functional evaluation of complete sciatic, peroneal, and posterior tibial nerve lesions in the rat Plast Reconstr Surg 1989 83 129 136 10.1097/00006534-198901000-00024 2909054

[B32] Barrera DA Zylstra E Lansbury PT Langer R Synthesis and RGD Peptide Modification of a New Biodegradable Copolymer: Poly (lactic acid-eo-lysine) J Am Chem Soc 1993 115 11010 11011 10.1021/ja00076a077

[B33] Seilheimer B Schachner M Regulation of neural cell adhesion molecule expression on cultured mouse Schwann cells by nerve growth factor EMBO J 1987 6 1611 1616 3608988 10.1002/j.1460-2075.1987.tb02408.x PMC553532

[B34] Bhang SH Lee T Yang HS La WG Han AM Kwon YHK Kim BS Enhanced nerve growth factor efficiency in neural cell culture by immobilization on the culture substrate Biochem Biophys Res Commun 2009 382 315 320 10.1016/j.bbrc.2009.03.016 19275890

[B35] Griffin JW Pan BH Polley MA Hoffman PN Farah MH Measuring nerve regeneration in the mouse Exp Neurol 2010 223 60 71 10.1016/j.expneurol.2009.12.033 20080088

